# Targeted therapies for vascular malformations

**DOI:** 10.3389/fmed.2024.1446046

**Published:** 2024-09-03

**Authors:** Gavin Kane, Israel Fernandez-Pineda

**Affiliations:** Children’s Health Ireland, Dublin, Ireland

**Keywords:** vascular, malformation, targeted, therapy, pediatric

## Abstract

Targeted medical therapies for the treatment of vascular malformations is an exciting and evolving area of research. As the identification of specific causative genetic mutations involved in vascular malformations becomes more accessible and inexpensive, the development of targeted therapies to address these genetic anomalies becomes all the more enticing. It is an excellent example of the potential of translational research where basic science discoveries are translated to clinical practise from ‘bench to bedside’. In this mini-review we aim to synopsise some of the recent studies published in this area with specific focus on the paediatric population. We also aim to highlight the growing demand for future research in the field to elucidate further the optimum duration of treatments, strategies for discontinuation, potential for combination of therapies and the effects of prolonged use of these medications.

## Introduction

Vascular anomalies comprise a diverse set of disorders. In 1996 the International Society for the Study of Vascular Anomalies (ISSVA) sought to give shape to this diversity by dividing lesions into two broad categories: Vascular Tumors and Vascular Malformations (1). The ISSVA has issued updates to their classification system, most recently in 2018, and the society continues to offer nuanced guidelines as our knowledge of the clinical and genetic features of vascular anomalies advances (2–4).

This mini-review will focus on vascular malformations. Vascular malformations are lesions that occur due to errors in the development of the vascular system (1, 3, 5–8). A vascular malformation can arise from any aspect of the vascular system including capillary, arterial, venous and lymphatic channels or a combination of these (1, 5). Vascular malformations can also be divided into ‘slow-flow’ lesions (capillary, venous and lymphatic malformations) and ‘fast-flow’ lesions (arteriovenous malformations) or a combination of both (8).

Vascular malformations can also form part of rare combined, complex and syndromic vascular anomalies (6). Some of these rare syndromes include (1) Klippel-Trenaunay Syndrome (KTS), (2) Congenital lipomatous overgrowth, vascular malformations, epidermal nevi and skeletal anomalies (CLOVES) syndrome and (3) Megalencephaly-capillary malformation-polymicrogyria/Macrocephaly-capillary malformation (MCAP/MCM) syndrome (4) Capillary malformation of the lower lip, Lymphatic malformation of the face and neck, Asymmetry of face and limbs, Partial/generalized Overgrowth (CLAPO) syndrome (6, 9). As genetic causes for these syndromes have been identified CLOVES, KTS, MCAP/MCM and CLAPO are now grouped together as PIK3CA-related overgrowth spectrum (PROS) (9, 10). Treatment of these syndromes will be discussed in more detail later in the review. Clinical complications such as chronic pain, disfigurement, recurrent infections, coagulopathies, organ dysfunction and death can occur secondary to vascular malformations (2). Vascular malformations are thought to be congenital in nature but they may alter secondary to trauma, infection or hormones (1). Comprehensive management of vascular malformations requires multi-disciplinary input from specialties such as surgery, radiology, dermatology, haematology-oncology, pathology and geneticists in collaboration with basic, translational and clinical research (3). Vascular malformations can be treated with various techniques the reader may be familiar with such as laser therapy for capillary malformations, sclerotherapy for macrocystic lymphatic malformations, and surgical debulking (1).

Traditional teaching suggests that vascular malformations are stable and non-proliferative (1). However, recent advances in targeted medical therapies have challenged this dogma. Medical therapies are now being used to target cellular pathways involved in abnormal vascular proliferation and growth which have been successful in treating vascular malformations (2). The focus of this review article will be on these targeted medical therapies utilized in the management of vascular malformations.

**Figure 1:**
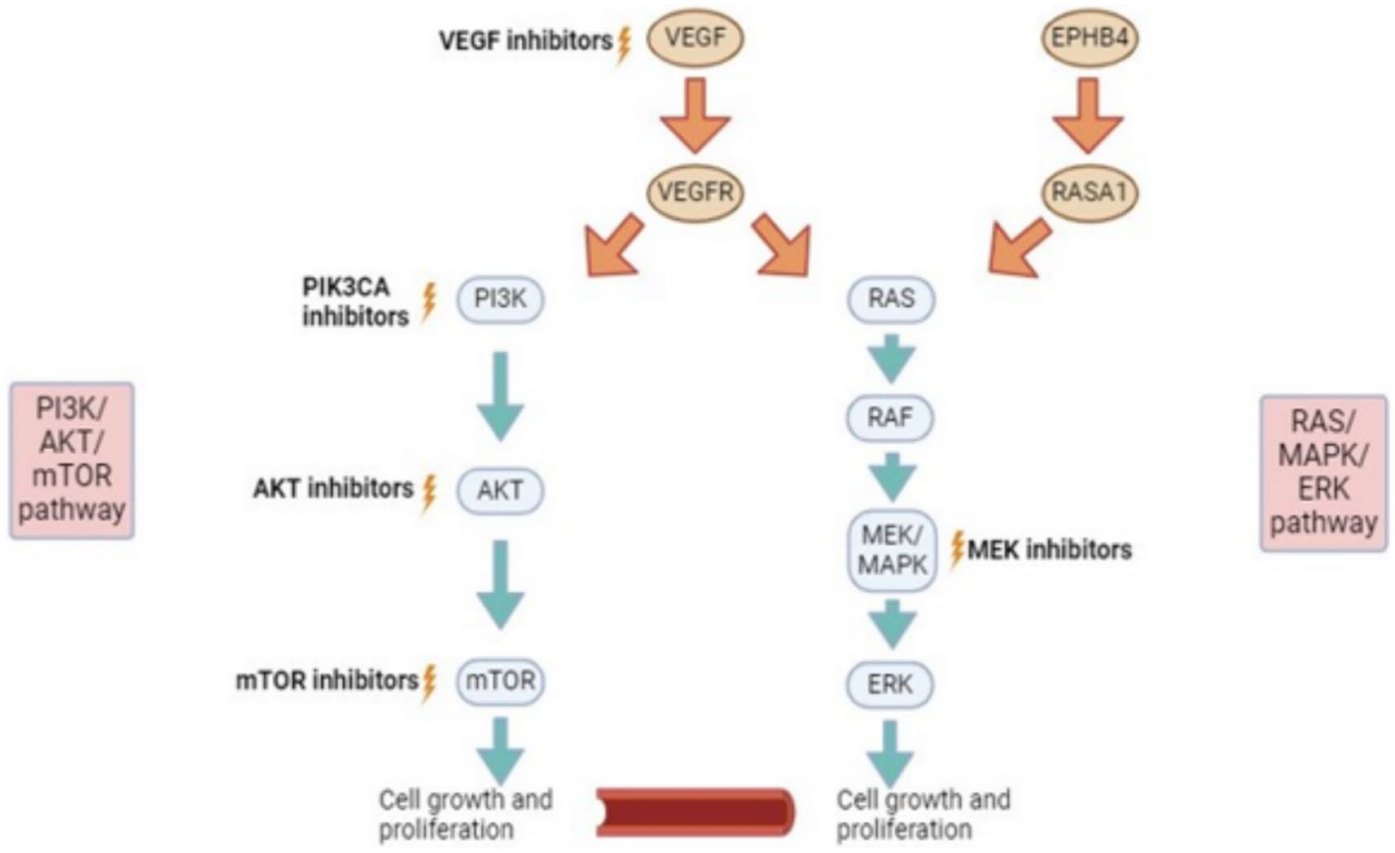
Signalling pathways and potential for targeted therapies. VEGF, vascular endothelial growth factor. EPHB4, ephrin type-B receptor 4; RASA1, RAS P21 protein activator 1 (a protein coding gene); PI3K, phosphatidylinositol-3 kinase; AKT (PKB), protein kinase b; mTOR, mammalian target of rapamycin; RAS, rat sarcoma virus; RAF, rapidly accelerated fibrosarcoma; MEK/MAPK, mitogen-activated protein kinase; ERK, extracellular signal-regulated kinase.

Before proceeding to discuss the relevant medical therapies, it is essential to look at the cell signaling pathways the medications are attempting to alter:

Current medical therapies can target three different signaling pathways (1) PI3K/AKT/mTOR pathway (2) RAS/MAPK/ERK pathway and (3) VEGF pathway (Figure 1). In short, as well as other important functions, these pathways lead to vascular cell proliferation, growth and differentiation (11). Almost all of the targets of the medications are proteins.

Discoveries in the recent past regarding causative genomic mutations have expanded our medical therapeutic options (3). Please see Table 1 for detected genetic mutations linked to certain vascular malformations.

**Table 1:** Types of vascular malformations and detected genetic mutations.

Pathway	Vascular malformation	Genetic mutation
	Common (cystic) lymphatic malformations	PIK3CA
	Generalised lymphatic anomaly (GLA)	PIK3CA
	Venous malformations	TEK (TIE2) / PIK3CA
PI3K/AKT/mTOR Pathway	Klippel-trenaunay syndrome	PIK3CA
	CLOVES syndrome	PIK3CA
	PIK3CA-related overgrowth spectrum (PROS)	PIK3CA
	Megalencephaly-capillary malformation-polymicrogyria (MCAP) syndrome	PIK3CA
	Proteus syndrome	AKT1
	Blue rubber bleb nevus syndrome	TEK (TIE2)
	Arteriovenous malformations	KRAS/MAP2K1, BRAF
RAS/MAPK/ERK pathway	Capillary malformation-arteriovenous malformation	RASA1, EPHB4
	Hereditary haemorrhagic telangiectasia	RASA1
	Kaposiform lymphangiomatosis	NRAS

PIK3CA, phosphatidylinositol-4,5-bisphosphate 3-kinase catalytic subunit alpha; TEK, TEK receptor tyrosine kinase (also called TIE2 gene); KRAS, kristen rat sarcoma viral oncogene; BRAF, v-raf murine sarcoma viral oncogene homolog B1; NRAS, neuroblastoma RAS viral oncogene homolog; CLOVES, Congenital lipomatous overgrowth, vascular malformations, epidermal nevi and skeletal anomalies.

## Targeted therapies for vascular malformations

### mTOR inhibitor (example: Sirolimus)

Sirolimus takes its action on the PI3K/AKT/mTOR pathway (Figure 1). Sirolimus is a potent inhibitor of mTOR (3). Sirolimus has reproducibly demonstrated ability to inhibit lymphangiogenesis in three separate model systems, including wound healing (11), embryonic development (12), tumor formation and metastasis (13).

The reader may be familiar with Sirolimus, also known as Rapamycin, due to its use as a preventor of kidney transplant rejection (2). Sirolimus has also been used to reduce the size of lesions in diseases such as tuberous sclerosis and lymphangioleiomyomatosis (14). First publication of its use in vascular malformations can be seen in the case series published by Hammill et al. in (1), in which 6 patients were offered Sirolimus for the treatment of complex vascular anomalies. In this series, 5 out of 6 patients studied had vascular malformations (1). All patients had been treated with previous medical and surgical therapies which had failed and oral Sirolimus was offered on compassionate grounds (1). All 6 patients in this study had a significant positive response to Sirolimus despite being previously refractive to other treatments (1).

A phase II clinical trial published by Adams et al. in 2016 bolstered the foundations of the aforementioned study. 61 patients with various vascular anomalies were enrolled in the study and 46 patients completed all 12 courses of the protocol Sirolimus therapy (2). The protocol therapy commenced with a Sirolimus dose of 0.8 mg/m (2) given orally twice daily. Trough Sirolimus levels were sustained between 10 and 15 ng/mL. Each treatment course lasted 28 days and the protocol duration was 12 courses per patient (2). Many of the patients in the study had vascular malformations rather than vascular tumors (2). One of the issues that plagues research in this field is defining an optimal response measure to the medical therapies. Currently this definition of optimal response does not exist. Therefore, Adams et al. in 2016 used 3 criteria to assess response: radiological response, functional impairment score and health related quality of life (2). The trial demonstrated an overall response rate of 83% at 6 months treatment and 85% at 1 year of treatment (2). With reference to the updated ISSVA classification system several disease entities involving vascular malformations had 100% partial response to Sirolimus at end of treatment at 6 months and end of treatment at 12 months in this study such as (1) GLA, (2) capillary-lymphatic venous malformation (CLVM), (3) Phosphatase and tensin homolog (PTEN)/arterial venous malformation, and (4) venous lymphatic malformation (2). Gorham syndrome also had 100% partial response at the halfway point of the study (i.e., at the end of treatment course 6 of 12) (2). The patients in this study had significant improvement in quality of life and in clinical symptoms regardless of whether they had improvement in their radiological response (2). This finding has been reproduced in further studies such as Harbers et al. (15) in which 68 patients were assessed (33 paediatric patients and 35 adults). They found that only 35% patients receiving Sirolimus had an improvement in the size of their lesion on imaging but 74% patients experienced an improvement in clinical symptoms (15).

In recent years, Sirolimus has also been utilised in the management of Kaposiform Lymphangiomatosis (KLA), a rare subtype of the vascular malformation Generalized Lymphatic Anomaly (GLA) (2, 16–22). KLA is a multifocal, invasive vascular malformation that most commonly presents with respiratory symptoms and haemorrhagic effusions in children (16). Surgical treatment for KLA is frequently not viable due to the diffuse nature of the disease and the affected locations (23). KLA has a poor overall survival rate in comparison to GLA, with studies suggesting survival rates as low as 35% (16).

The pathogenesis of KLA is not completely understood at present. However, some knowledge of its pathogenesis has been established in recent studies which detected mutations in Q61R of the NRAS (neuroblastoma RAS viral oncogene homolog) gene in patients with KLA (24, 25). Moreover, there are *in-vitro* studies that suggest NRAS mutations are linked to cell proliferation through both the AKT/PI3K and MAPK pathways (26).

Due to the rarity of the disease, combining the results of multiple centres treating KLA with Sirolimus is a sensible option. Using the results from 7 of their own patients and the results from 7 other studies, Zhou et al. (16), report outcomes for a total of 24 patients with KLA treated with Sirolimus (2, 16–22). 14 patients (58%) obtained a partial response, 6 (25%) had stable disease and only 4 (16.7%) suffered disease progression (2, 16–22). The results suggest that Sirolimus can certainly play a role in the treatment of KLA but it is not the panacea for this complex disease as no patients across all the aforementioned studies achieved complete response.

In light of the reference to KLA above, it is important to note that Sirolimus has been successfully utilised in combination with surgical and interventional radiological procedures to improve overall patient outcomes as well as a targeted therapy on its own (2, 27–30).

### Dosing

Dose regimens and drug monitoring may vary between institutions but the most commonly implemented regimen for Sirolimus follows that used in the 2016 phase II clinical trial (2). The medication is taken orally. Initial dosing is 0.8 mg/m (2) per dose, administered twice daily at approximately 12-h intervals. Subsequent dosing adjustments are made in order to maintain a goal drug trough level of 10–15 ng/mL (1, 2).

However, it has been suggested that lower doses of Sirolimus can be still efficacious in treating vascular malformations and the lower trough levels may reduce the risk of side-effects (3, 15). For example, a recent study involving 33 paediatric and 35 adult patients with slow-flow malformations suggested that Sirolimus was effective in treating 79.1% patients at lower trough levels of 4-10 ng/mL (15).

On the back of a study by Mizuno et al. in 2017, it has been suggested that neonates (0–1 month) should be commenced on a lower dose of 0.4–0.45 mg/m (2) per dose, also targeting a trough concentration level of 10–15 ng/mL (31). This is due to the lower levels of Cytochrome-P450, 3A (CYP3A) in the intestines and liver of this age group (31). The drug levels should also be monitored early and frequently in this age category (every 3 days) to avoid supratherapeutic levels until a stable dose has been established (31).

In all patients, Sirolimus levels must be checked regularly to assess trough levels (1). There have been recommendations in the adult literature in reference to Sirolimus use in Lymphangioleiomyomatosis (a rare lung disease) to check levels 3 weeks after commencing therapy or after dose changes and then 3 monthly thereafter (32).

### Complications/side-effects

The most common side-effects include asthenia, mucositis, diarrhoea, acne, lymphoedema, hyperlipidaemia, headache, bone marrow suppression and risk of infection (1–3, 8).

Hammill et al. 2011 contended that no significant infections occurred in their patient group that could be directly attributable to Sirolimus treatment (1). One of their patients had to discontinue Sirolimus treatment due to issues with mucositis (1).

The most common adverse effect in the study by Adams et al. in 2016 was related to blood/bone marrow and occurred in 27% of their patients (2).

Other more rare but significant complications can include: pneumonitis, potential male infertility, issues with wound healing, capillary leak syndrome and ovarian cysts (3).

Due to the immature immune system in neonates and infants, for patients in this category taking Sirolimus, formal testing for immune dysfunction is recommended (3).

The adverse effects experienced by adults and children taking Sirolimus appear to be different. This is born out in the study by Harbers et al. (15) which suggested that the paediatric population experienced a grade I-IV adverse event related to Sirolimus less often, with statistical significance, than the adult population. The Common Terminology Criteria for Adverse Events version 3.0 (CTCAE v3.0) grades adverse events from I to V (33).

### Duration of therapy

There is no clearcut data/evidence to generate guidelines for optimal duration of therapy for Sirolimus use in vascular malformations. Some studies suggest that the majority of their patients become symptomatic again after stopping Sirolimus whereas others have suggested that only a third of their patients required reinstitution of therapy (8, 15). At present, treatment duration differs on a case by case basis and for some patients, it may be indefinite. Further longitudinal data is needed to give physicians and their families the armamentarium to make accurate decisions about duration of treatment (3).

### AKT inhibitors (example: Miransertib)

These medications target AKT which is a protein upstream of MTOR on the PI3K/AKT/mTOR pathway (Figure 1) (3). Inhibition of AKT has demonstrated efficacy *in-vitro* on patient derived cells received from patients with vascular malformations as well as other vascular anomalies (34). A study by Le Cras et al. (35) in 2020 displayed constitutive phosphorylation of AKT in patients with CLVMs and demonstrated inhibition of the proliferation of these cells by AKT inhibitors. Phosphorylation has a crucial role in cell signaling and refers to the addition of a phosphoric acid group to a protein, thus changing its structure (36).

However, prospects for the benefit of AKT inhibitors in the treatment of vascular malformations were dampened by results of the MOSAIC study published in 2022 (37). In this study, the AKT inhibitor Miransertib was deemed inefficacious in treating patients with PROS (37). Efficacy was also not demonstrated for treatment of patients with Proteus Syndrome, another overgrowth syndrome associated with hamartomas and vascular malformations (37).

### PIK3CA inhibitors (example: Alpelisib)

PIK3CA inhibitors target PI3K which is also upstream of MTOR on the PI3K/AKT/mTOR pathway (Figure 1). Alpelisib is a PIK3CA inhibitor that has Food and Drug Administration (FDA) approval for use in patients with breast cancer (3). With respect to vascular malformations, a PIK3CA inhibitor was used for patients with a severe form of CLOVES syndrome in 2015 (38). There were no significant side-effects noted from the medication in the patients involved other than mild hyperglycaemia (38). All patients involved in the study had symptom improvement and radiological and clinical decrease in disease burden (38). It is postulated that PIK3CA inhibitors act by reducing AKT phosphorylation and decreasing mTORC1 activation (3). The medication may offer more complete blockage of AKT in comparison to mTOR inhibitors (Sirolimus) and therefore, may be particularly beneficial for those who have had insufficient or no response to Sirolimus (3).

In August 2023, the EPIK-P1 study of the compassionate use of Alpelisib PIK3CA inhibitor for 57 patients with severe/life-threatening PROS was published (39). The drug was given FDA approval on the back of this study. The study included both paediatric patients >2 years of age (68.4%) and adults (31.6%). In this study, the agreed threshold of clinical response was a reduction in the sum of target lesion/s volume of greater than 20% from an index date (39). There were 31 patients on Alpelisib treatment that had imaging available at index start of treatment and at week 24 of treatment and 74.2% of these had a reduction in sum of the measurable target lesion/s (39). At the time of data cutoff, no patient had suffered radiological disease progression (39).

Independent of radiological response, all 57 patients in the study had improvement in PROS-related symptoms and signs over the study period (39). Almost 80% of the patients in the study had symptom improvement related to a vascular malformation (39).

The EPIK-P1 study did not collect data on specific lesion types so information regarding lesion-specific response to Alpelisib was not obtained (39). Therefore, for the purpose of this review article, it is not possible to pinpoint specific vascular malformations and their response to PIK3CA inhibitors. In a study published in Nature in 2018, Alpelisib was successfully used to treat 17 patients (14 children and 3 adults) with PROS with a response rate of 100% (38).

To further elucidate the efficacy, pharmacokinetics and safety of Alpelisib use in paediatric and adult cases of PROS, there is a randomized, double-blinded, placebo-controlled prospective study ongoing named EPIK-P2 NCT04589650 which is actively recruiting patients (39).

### Dosing

In the studies available the dose offered was 50 mg for children and 250 mg once daily for adults (38–41).

### Complications/side-effects

The side-effects appear to be mild (38, 39) 72.2% adults experienced side-effects but only 23.1% of the paediatric population experienced side-effects in EPIK-P1 study (39). No patient discontinued use of Alpelisib due to side-effects (39). The most common side-effects experienced were diarrhoea, hyperglycaemia and aphthous ulcers (39). Similarly, in Venot et al’s. (38) study, the most common side-effects were hyperglycaemia and gastrointestinal symptoms.

### MEK inhibitors (example: Trametinib)

MEK/MAPK is an enzyme that forms part of the RAS/MAPK/ERK pathway (Figure 1). MEK inhibitors are FDA-approved as targeted therapies for malignant melanoma (3).

Several small studies have demonstrated that these medications can prove beneficial in patients with mutations in the RAS/MAPK/ERK pathway leading to specific vascular malformations (3). Lekwuttikarn et al. (42) describe the use of a MEK inhibitor for a patient with a large arteriovenous malformation (AVM) on the trunk. The lesion responded successfully in terms of size, color and warmth (42). In 2020 in the United States, a patient with a KRAS mutation was treated with Trametinib for a spinal AVM and was found to have decreased arterial inflow to the malformation (43). As well as this, Trametinib was used successfully to treat a patient with capillary malformation-AVM syndrome and resultant high-output cardiac state (44). The patient had a known mutation in EPHB4 which is a receptor tyrosine kinase upstream from the RAS/MAPK/ERK pathway (Figure 1) (45).

Dori et al. in 2020, treated three patients with severe lymphatic disorders with Trametinib resulting in significant symptomatic improvement on top of a successful radiological response (46). These 3 patients had mutations related to the RAS/MAPK/ERK pathway (Figure 1). One patient had ARAF (Serine/threonine-protein kinase A-Raf) mutation, one had KLA (Klotho-alpha) and CBL (Casitas B-lineage Lymphoma) mutation and the final patient had SOS1 (SOS Ras/Rac guanine nucleotide exchange factor 1) mutation (46). The ideal dosing regimen for Trametinib in the paediatric population for vascular malformations needs to be further investigated.

### Complications/side-effects

The most common side-effects experienced with Trametinib use are: an acneiform rash, palmar-plantar erythrodysesthaesia and paronychia. Supportive care is usually sufficient to address these issues (3).

## Discussion

As genetic testing becomes more accessible and inexpensive, genetic analysis is beginning to become part of the workup upon diagnosis of certain vascular anomalies (3). If a genetic mutation is identified this could potentially offer a targeted medical treatment for the patient. Targeted medical therapies for vascular anomalies are excellent examples of the benefit of translational research in which basic science is translated to clinical practice from the ‘bench to the bedside’.

Due to the fact that it is most often not possible to eradicate a vascular malformation entirely, a definition of the optimal response to medical therapy is an area that requires further research and standardization. Previous studies have used a combination of the following points to delineate treatment response: reduction in malformation size, stabilization of growth, correction of coagulation abnormalities, reduced lymphatic leakage, decreased rate of infection and improved quality of life (3, 15).

A significant limitation of previous studies in this field is the inclusion of numerous disease entities in their analysis (2, 3). Also, in certain studies such as Adams et al. in 2016, all of the patients had received different treatments prior to receiving novel targeted therapy including surgery, medications and interventional radiology meaning that the patients could not be defined as ‘common denominators’ at the beginning of the study (2). The side-effect profile of the therapy must also factor in the decision-making for its use. This is most relevant in the case of Sirolimus. It is noteworthy that in certain studies up to three quarters of patients suffered adverse effects and one third of these were grade III or IV in severity (47).

As the role of targeted medical therapies for vascular malformations is likely to expand, current research gaps need to be filled in regards to: optimum duration of therapies, options for discontinuation of medications, safety issues with long-term use of medications and potential late complications of prolonged use (3).

Although there have been some very promising advances in this field, especially with respect to Sirolimus, the potential for future developments is extensive. There is growing need for standards of practise to be generated and outcomes of future research studies and trials is what is required for this to come to fruition (3).
